# Horizontal Transfer of the *Salmonella enterica* Serovar Infantis Resistance and Virulence Plasmid pESI to the Gut Microbiota of Warm-Blooded Hosts

**DOI:** 10.1128/mBio.01395-16

**Published:** 2016-09-06

**Authors:** Gili Aviv, Galia Rahav, Ohad Gal-Mor

**Affiliations:** a The Infectious Diseases Research Laboratory, Sheba Medical Center, Tel-Hashomer, Israel; b Department of Clinical Microbiology and Immunology, Tel Aviv University, Tel Aviv, Israel; c Sackler Faculty of Medicine, Tel Aviv University, Tel Aviv, Israel

## Abstract

*Salmonella enterica* serovar Infantis is one of the prevalent salmonellae worldwide. Recently, we showed that the emergence of *S*. Infantis in Israel was facilitated by the acquisition of a unique megaplasmid (pESI) conferring multidrug resistance and increased virulence phenotypes. Here we elucidate the ecology, transmission properties, and regulation of pESI. We show that despite its large size (~280 kb), pESI does not impose a significant metabolic burden *in vitro* and that it has been recently fixed in the domestic *S*. Infantis population. pESI conjugation and the transcription of its pilus (*pil*) genes are inhibited at the ambient temperature (27°C) and by ≥1% bile but increased under temperatures of 37 to 41°C, oxidative stress, moderate osmolarity, and the microaerobic conditions characterizing the intestinal environment of warm-blooded animals. The pESI-encoded protein TraB and the oxygen homeostasis regulator Fnr were identified as transcriptional regulators of pESI conjugation. Using the mouse model, we show that following *S*. Infantis infection, pESI can be horizontally transferred to the gut microbiota, including to commensal *Escherichia coli* strains. Possible transfer, but not persistence, of pESI was also observed into Gram-positive mouse microbiota species, especially *Lactobacillus reuteri*. Moreover, pESI was demonstrated to further disseminate from gut microbiota to *S. enterica* serovar Typhimurium, in the context of gastrointestinal infection. These findings exhibit the ability of a selfish clinically relevant megaplasmid to distribute to and from the microbiota and suggest an overlooked role of the microbiota as a reservoir of mobile genetic elements and intermediator in the spread of resistance and virulence genes between commensals and pathogenic bacteria.

## IMPORTANCE

Plasmid conjugation plays a key role in microbial evolution, enabling the acquisition of new phenotypes, including resistance and virulence. *Salmonella enterica* serovar Infantis is one of the ubiquitous salmonellae worldwide and a major cause of foodborne infections. Previously, we showed that the emergence of *S*. Infantis in Israel has involved the acquisition of a unique megaplasmid (pESI) conferring multidrug resistance and increased virulence phenotypes. Recently, the emergence of another *S*. Infantis strain carrying a pESI-like plasmid was identified in Italy, suggesting that the acquisition of pESI may be common to different emergent *S*. Infantis populations globally. Transmission of this plasmid to other strains or bacterial species is an alarming scenario. Understanding the ecology, regulation, and transmission properties of clinically relevant plasmids and the role of the microbiota in their spreading offers a new mechanism explaining the emergence of new pathogenic and resistant biotypes and may assist in the development of appropriate surveillance and prevention measures.

## INTRODUCTION

*Salmonella enterica* is a Gram-negative, facultative intracellular human and animal pathogen posing a major public health concern worldwide (1). *Salmonella* infection is one of the most common causes of foodborne illnesses and a major cause of diarrheal diseases in developed and developing countries. A total of 93.8 million cases of gastroenteritis due to nontyphoidal *Salmonella* (NTS) infection are estimated annually, resulting in approximately 155,000 deaths (2). The distinct species *S. enterica* includes more than 2,600 different serovars, which share high sequence similarity (3, 4).

*S. enterica* serovar Infantis has recently been emerging worldwide. In the United States, *S*. Infantis was recently ranked sixth in the prevalence order of *Salmonella* serovars (5) and in the European Union, *S*. Infantis was ranked third, after *S. enterica* serovars Enteritidis and Typhimurium (6). In Israel, during 2008 to 2015, *S*. Infantis was the most predominant serovar, responsible for over 30% of all salmonellosis cases in humans (7, 8). Our previous work reported that the rapid emergence of an endemic *S*. Infantis clone was facilitated by the acquisition of a novel megaplasmid (~280 kb), termed pESI. We showed that this conjugative plasmid confers resistance to multiple antimicrobial drugs, heavy metals, and disinfectants and enhances virulence-associated phenotypes and the pathogenicity of its bacterial host (7). Recently, the emergence of a different *S*. Infantis strain carrying a pESI-like plasmid with an extended-spectrum β-lactamase (ESBL)-producing gene was identified in Italy (9), suggesting that the acquisition of pESI may be common to geographically distinct emerging *S*. Infantis populations.

One of the main mechanisms by which bacteria become resistant to antibiotics and gain new phenotypes is the acquisition of genes via plasmid conjugation (10, 11). Thus, conjugation plays a key role in horizontal gene transfer (HGT) and bacterial genome plasticity. During this process, genetic material is transferred from a donor bacterial cell to a recipient bacterium through a bridge-like connection known as the conjugative pilus. Conjugation in Gram-negative bacteria is mediated by the type IV secretion system (T4SS), comprising 12 to 30 proteins that are assembled into a large exporting machinery, spanning the inner and outer membranes and involved in substrate transport and pilus biogenesis (12). The genes for the conjugative machinery are encoded by autonomously replicating plasmids or by chromosomally encoded integrative and conjugative elements (ICEs) (13).

Here we report the complete fixation of pESI in the domestic *S*. Infantis population, identify environmental cues and bacterial regulators that control pESI conjugation, and elucidate its metabolic burden and copy number, in addition to stages in the modular formation of pESI. Additionally, we show that pESI is capable of disseminating in the gut of warm-blooded animals and demonstrate its interspecies transfer to the mouse microbiota and from there to another pathogen in the context of infection. These results highlight the potential role of the gut microbiota as an intermediator in the spread of resistance, metabolic, and virulence genes in nature.

## RESULTS

### Stages in the modular formation of pESI and its prevalence in the population.

**FIG 1:**
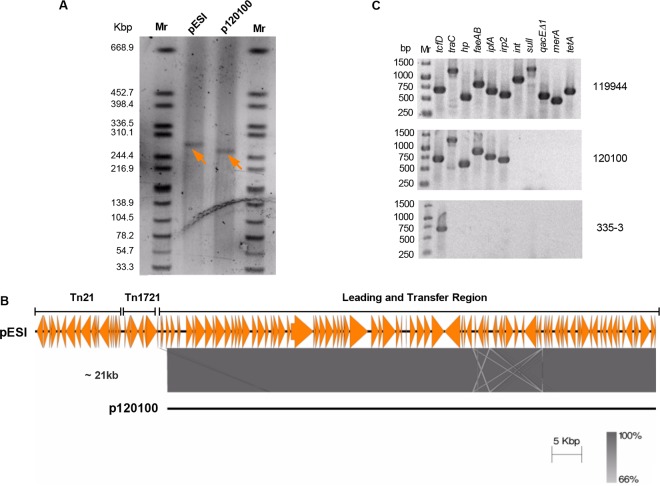
A modular formation of pESI. (A) Plasmid linearization using S1 nuclease followed by PFGE analysis was performed to determine the plasmid size of p120100 found in a 2008 *S*. Infantis food isolate (120100) in comparison to pESI. Linearized plasmids are indicated by arrowheads. (B) Pairwise comparison of a DNA fragment of about 120 kb corresponding to the leading and transfer region from pESI and p120100 was performed using the Easyfig tool. The colored bar indicates sequence homology between the overlapped regions. A region of about 21 kb containing the MGEs Tn*21*-like and Tn*1721*-like, which are absent from p120100, is indicated. (C) PCR analysis of chromosomal (*tcfD*), pESI backbone-carried (*traC*, *hp*, *faeAB*, *ipfA*, and *irp2*), and MGE-carried (int, *sulI*, *qacEΔ1*, *merA*, and *tetA*) genes. Template DNA was extracted from *S*. Infantis isolates 119944 (harboring pESI), 120100 (harboring p120100), and 335-3 (a plasmidless preemergent strain).

When we first reported the sequence and the genetic organization of pESI, it had no homologs in the databases, and although its transfer region is similar to the one of the IncI R64 plasmid (AP005147), it was unclear how pESI has evolved (7). To gain insights into the formation of pESI, we sought to identify related plasmid derivatives that can shed light on its assembly process. Analysis of the XbaI pulsed-field gel electrophoresis (PFGE) profiles of 87 *S*. Infantis isolates that were collected in Israel between 1970 and 2009 (8) led to the identification of a 2008 food isolate (no. 120100) that had a plasmid similar to pESI, differing by only one XbaI fragment (data not shown). Plasmid linearization using S1 nuclease of isolate 120100 followed by PFGE showed the presence of a plasmid (designated here p120100) that was 21 kb smaller than pESI (Fig. 1A). Whole-genome sequencing of strain 120100 confirmed that p120100 has the same backbone as pESI, but it lacks the mobile genetic elements (MGEs) Tn*21*-like and Tn*1721*-like located upstream from the leading and transfer region of pESI (Fig. 1B) and an ~1-kb class I integron. PCR amplification using primers from genes carried on pESI confirmed these results and showed the presence of the genes carried on the backbone (*traC*, hypothetical protein-encoding gene [*hp*], *faeAB*, *ipfA*, and *irp2*) but not the genes carried on the MGEs Tn*21*-like (*merA*, *sulI*, and *qacE*Δ*1*) and Tn*1721*-like (*tetA*) and the class I integron (*int*) (Fig. 1C). Thus, p120100 provides an evolutionary snapshot of the modular formation process of pESI, suggesting that the insertion of Tn*21*-like, Tn*1721*-like, and the class I integron into an ancestral p120100 (or similar) plasmid resulted in the formation of the mature pESI.

Previously, we showed that while *S*. Infantis strains that were isolated in Israel before 2007 (*n* = 16) did not contain the pESI megaplasmid, 82% (58/71) of the *S*. Infantis strains that were collected between 2007 and 2009 already harbored pESI (8). In order to gauge the spread of pESI among the domestic (Israeli) *S*. Infantis population, 48 isolates, randomly collected during 2014 from different sources (16 from human origin, 8 from food and 24 from poultry) were subjected to PCR analysis. Interestingly, all of the tested isolates (48/48) were found to contain the pESI plasmid (see Fig. S1 in the supplemental material). These results indicate a different prevalence of pESI than the one found in 2009 (*P* = 0.0017) and suggest the fixation of pESI in the local *S*. Infantis population.

### Estimation of pESI copy number and metabolic burden.

**FIG 2:**
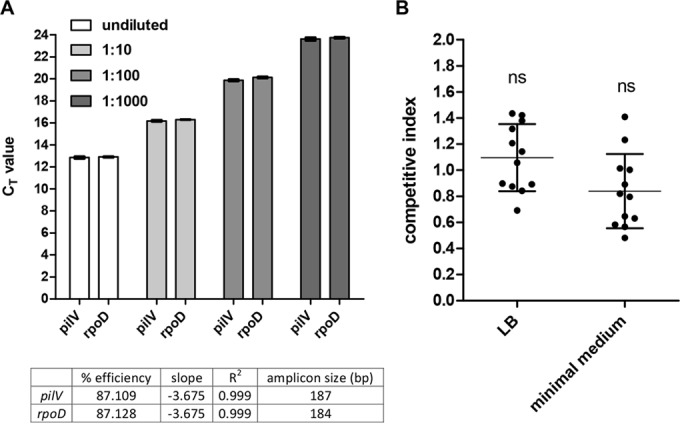
pESI is maintained in a single copy number and does not confer a significant metabolic burden *in vitro*. (A) Ten-fold serial dilutions of genomic DNA extracted from *S*. Infantis 335-3 harboring pESI were subjected to real-time PCR using specific primers of the pESI-carried gene *pilV* and the chromosome-carried gene *rpoD*. Bars represent the mean threshold cycle (*C_T_*) values and the standard deviation (SD) from three biological repeats. The *C_T_* values of each gene at the different dilutions were used to create a standard curve. The amplicon size (in base pairs), amplification efficiency (percentage), the slope of the standard curve, and the coefficient of determination (*R*^2^), showing the linearity of the standard curve, are indicated in the bottom table. (B) Competitive index of *S*. Infantis 335-3 carrying pESI and its isogenic strain lacking pESI. Both strains were grown together in LB and N-minimal medium for 24 h at 27°C. Competitive index values were determined as (335-3-pESI/335-3)_output_/(335-3-pESI/335-3)_input_. Dots represent the results of single (out of 12) independent competition experiments; the mean and SD are shown by the horizontal line and error bars, respectively. A D’Agostino and Pearson omnibus normality test confirmed Gaussian distributions, and a one-sample *t* test against a theoretical mean of 1 was used to determine statistical significance of the mean. ns, not significant (*P* > 0.05).

Taking into account the large size (~280 kb) of pESI and its high prevalence, we aimed to assess its potential metabolic burden. Since plasmid copy number contributes significantly to the metabolic cost of large plasmids (14), the pESI copy number in the *S*. Infantis host was determined using the relative quantification approach (15). The threshold cycle (*C_T_*) value of the single-copy, chromosomally carried gene *rpoD* was compared by quantitative real-time PCR (qRT-PCR) to the *C_T_* of a pESI-carried gene, *pilV* (coding for the minor pilin subunit of the conjugative pilus). Since *pilV* and *rpoD* share similar amplification efficiencies and amplicon sizes, the same *C_T_* value, which was obtained for both targets over a series of DNA dilutions (Fig. 2A), indicates a single copy number of pESI in the *S*. Infantis host.

To estimate the possible metabolic burden of pESI on its host, competition assays were conducted. *S*. Infantis 335-3, which harbors pESI, and equal CFU of its isogenic strain lacking this plasmid were grown together without antibiotic selection in rich LB broth and in minimal medium for 24 h. To reduce pESI conjugation during competition, the cultures were grown with vigorous shaking and at 27°C (see below). CFU enumeration postcompetition showed no significant difference in the abundance of the strain carrying pESI relative to the pESI-free strain (Fig. 2B). These results support the notion that despite its considerable size, pESI does not confer a significant metabolic burden *in vitro*, providing a possible explanation for its very high prevalence among the *S*. Infantis population.

### pESI conjugation and pilus gene transcription are induced under microaerobic, physiological temperature, and oxidative stress conditions.

**FIG 3:**
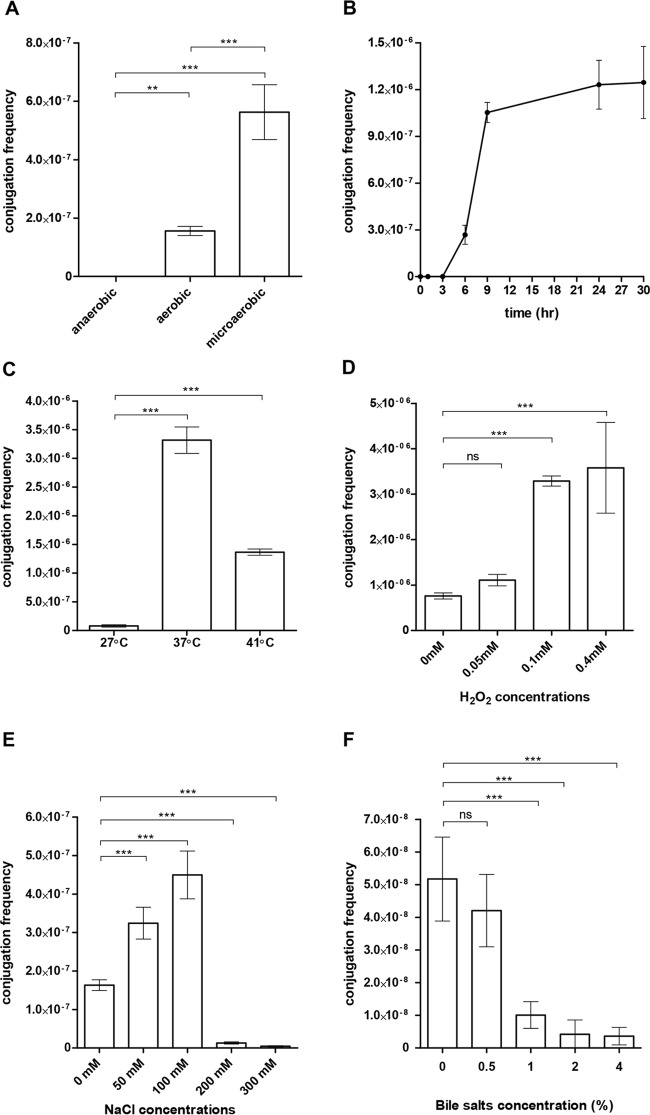
pESI conjugation is increased in response to microaerobiosis, physiological temperature, and moderate osmolarity. The pESI conjugation frequency (obtained transconjugants/donor CFU) between *S*. Infantis 119944 (donor) and *E. coli* ORN172 (recipient) was determined under (A) different oxygen conditions, (B) over time under a microaerobic environment, and at different (C) temperatures, (D) hydrogen-peroxide concentrations, (E) sodium chloride concentrations, and (F) bile salt concentrations. Bars show the means and SDs from at least four independent mating experiments. One-way analysis of variance (ANOVA) with Tukey’s (for panels A and C) or Dunnett’s (for panels D to F) multiple comparison tests was implemented to determine statistical significance. ns, not significant; **, *P* < 0.001; ***, *P* < 0.0001.

Our next efforts were focused on the transmission properties of pESI and the way its conjugation is regulated. Mating between *S*. Infantis 119944 (donor) and *Escherichia coli* K-12 strain ORN172 (recipient) was studied over time and under various physiologically relevant conditions. These experiments showed that pESI conjugation frequency (number of transconjugants per donor cell) diminishes under anaerobic conditions but increases under aerobic conditions, and even more so under microaerobic conditions (Fig. 3A). Under microaerobiosis at 37°C, we were able to detect the appearance of *E. coli* transconjugants within 3 h of conjugation that has reached a constant maximal frequency (up to 1 × 10^−6^) after 9 h (Fig. 3B). Besides microaerobiosis, temperature was also found to greatly affect pESI conjugation, which was significantly increased at 37 or 41°C relative to 27°C (Fig. 3C). Interestingly, oxidative stress, which was previously shown to affect conjugation of other conjugative plasmids (16), was found to moderately increase the conjugation frequency of pESI in a hydrogen peroxide dose-dependent manner (Fig. 3D).

Furthermore, we measured pESI conjugation under different osmolarity (sodium chloride) conditions. In the human digestive tract, the sodium concentration ranges from 20 mM in the stomach to about 100 mM in the jejunum (17). Similar values were also reported for the luminal fluid in the small intestine of broiler chickens, where the sodium concentration ranges from 67 to 118 mM across segments (18). Therefore, pESI conjugation was tested in the presence of 0, 50, 100, 200, and 300 mM NaCl. We found that the conjugation frequency is maximal at 100 mM NaCl but significantly inhibited at higher concentrations (200 to 300 mM) (Fig. 3E) as well as by the presence of ≥1% bile salts (Fig. 3F).

**FIG 4:**
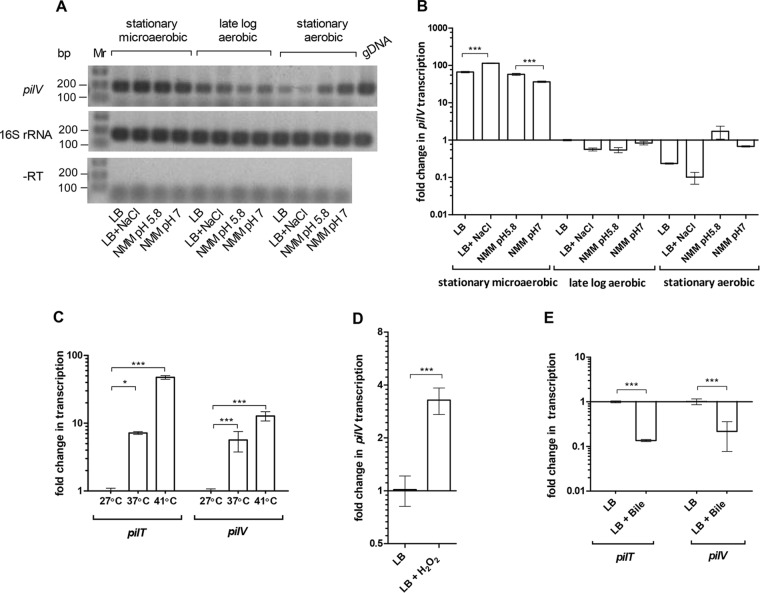
The transcription of pESI pilus is induced in response to microaerobiosis and physiological temperature and repressed by bile. (A) Semiquantitative reverse transcription-PCR analyses of *pilV* and 16S rRNA transcripts. RNA was extracted from *S*. Infantis 119944 cultures grown in LB, LB supplemented with 0.3 M NaCl, N-minimal medium (NMM) at pH 5.8, and N-minimal medium at pH 7.0. Templates of genomic DNA (gDNA) or purified RNA without reverse transcriptase treatment (−RT) were included as positive and negative controls, respectively. (B) qRT-PCR shows the fold change in *pilV* transcription under the same growth conditions as in panel A relative to the transcription of *pilV* in LB late-logarithmic culture grown under aerobic conditions. (C) Fold change in the transcription of *pilV* and *pilT* grown in LB under microaerobic conditions at 37 and 41°C relative to 27°C. (D) RNA was extracted from *S*. Infantis 119944 grown in LB and in LB supplemented with 0.1 mM H_2_O_2_ under microaerobic conditions at 37°C. qRT-PCR analysis was conducted to determine the fold change in the transcription of *pilV*. (E) Fold change in the transcription of *pilV* and *pilT* grown under microaerobic conditions at 37°C in LB supplemented with 4% bile salts (sodium choleate). All RT-PCR results show the mean and SD from three to six biological repeats. Two-tailed *t* test (B, D, and E) or one-way ANOVA with Tukey’s multiple comparison test (C) was implemented to determine statistical significance. *, *P* < 0.05; ***, *P* < 0.0001.

To better understand the regulation of the pESI conjugation on the transcriptional level, reverse transcription–real-time PCR was used to measure the expression of *pilV* and *pilT*, both involved in the formation of the type IV pilus (19) under different growth conditions, including growth phases (stationary versus logarithmic), media (rich LB broth versus minimal medium), pH (5.8 versus 7.0), temperatures (27, 37, and 41°C), and the presence of bile and hydrogen peroxide. Consistent with the conjugation results, we found that *pilV* transcription is induced by more than 60-fold under microaerobic conditions at the stationary phase (Fig. 4A and B) and that *pilV* and *pilT* expression is induced at 37°C or 41°C, but not at the ambient temperature of 27°C (Fig. 4C). Similarly, *pilV* transcription was induced in the presence of 0.4 mM H_2_O_2_ (Fig. 4D). In contrast, *pilV* and *pilT* transcription was repressed in the presence of 4% bile (Fig. 4E). We concluded that pESI conjugation and pilus gene transcription are induced at physiological temperatures, under microaerobic and oxidative stress conditions, but repressed at the ambient temperature and in the presence of >1% bile.

### TraB and FNR are transcriptional regulators of pESI *pil* genes.

**FIG 5:**
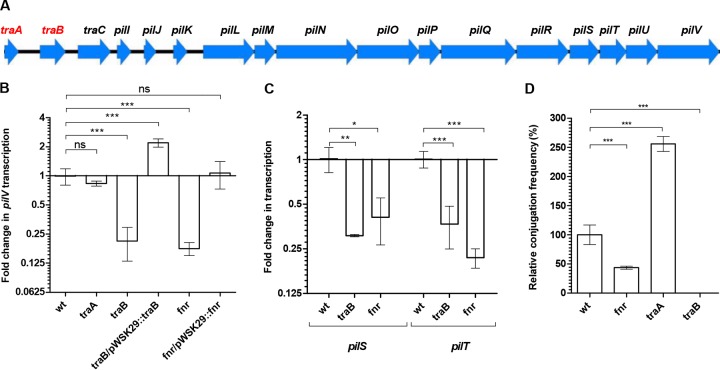
pESI conjugation and pilus transcription are regulated by TraB and FNR. (A) Gene organization of the *pil* operon carried on pESI. Arrowheads show the location and orientation of the different open reading frames (ORFs). Putative regulatory genes are shown in red. (B and C) RNA was extracted from cultures of *S*. Infantis 119944 (wild type [wt]) and its derivative mutants (*traA*, *traB*, and *fnr*) and *traB*/pWSK29::*traB* and *fnr*/pWSK29::*fnr* complemented strains grown in LB under microaerobic conditions at 37°C. qRT-PCR analyses was conducted to determine the fold change in the transcription of *pilV* (B) and *pilS* and *pilT* (C) in the indicated backgrounds relative to the wild-type strain. (D) pESI conjugation between *S*. Infantis strain 119944 or its *fnr-*, *traA-*, and *traB-*derived mutant strains and *E. coli* ORN172 were conducted on LB plates under microaerobic conditions. Results show the mean and SD from at least three biological repeats. One-way ANOVA with Dunnett’s multiple comparison test was implemented to determine statistical significance. *, *P* < 0.005; **, *P* < 0.001; ***, *P* < 0.0001; ns, not significant.

pESI sequence analysis revealed the presence of two putative regulatory genes—*traA* and *traB*—located upstream from the *traC* and *pil* genes (Fig. 5A). TraA, containing a helix-turn-helix DNA-binding domain, is similar to a bacterial regulator from the LuxR family (GI:476192203), and TraB is similar to the transcription termination factor NusG (GI:447136371). To further study the role of these putative regulators in controlling the transcription of the conjugation system in pESI, an in-frame deletion mutant of *traA* and *traB* was constructed in the *S*. Infantis 119944 background. Additionally, we screened for a possible regulatory role 10 global regulators previously reported to control conjugation of other plasmids (Lrp [20] and ArcA and ArcB [21]), to be involved in oxygen homeostasis (OxyR, SoxR, and FNR [22]), or to regulate key virulence phenotypes in *Salmonella* (RpoS, PhoP, OmpR, and Fur [23]).

qRT-PCR did not show a significant change in the transcription of *pilV* in the absence of Lrp, ArcA, ArcB, OxyR, SoxR, RpoS, PhoP, OmpR, Fur (data not shown), and TraA (Fig. 5B); however, the lack of TraB and FNR resulted in 4- and 7-fold decreases in the transcription of *pilV*, respectively, under microaerobic conditions. Ectopic expression of *traB* and *fnr* from a low-copy-number plasmid (pWSK29) restored *pilV* expression to levels similar to or even higher than those of the wild-type strain (Fig. 5B). Similar results in the absence of TraB and FNR were also obtained for the transcription of *pilS* and *pilT* (Fig. 5C). In agreement with these results, pESI conjugation in the absence of FNR and TraB was decreased by more than 2-fold and below the detection level, respectively (Fig. 5D). Moreover, downregulation of *pilV* and *pilS* expression in the absence of FNR was not observed under aerobic growth conditions (data not shown), consistent with the inactive form of FNR under aerobiosis (24).

Together, these results indicate that both FNR and TraB act as positive transcriptional regulators of the conjugative pilus genes and that they are required for optimal pESI conjugation. Interestingly, although a significant change in the *pilV* transcription in the absence of TraA was not observed (Fig. 5B), *traA* deletion led to a consistent 2.5-fold increase in pESI conjugation frequency (Fig. 5D), suggesting that TraA may act as a negative regulator of other conjugation genes or functions at the posttranscriptional level.

### pESI is transferred to the gut microbiota during *Salmonella* infection.

Having established that the transcription of the *pil* genes and pESI conjugation are both induced under physiological temperature, microaerobiosis, and moderate osmolarity, characterizing niches of the gastrointestinal (GI) tract in warm-blooded animals, we hypothesized that horizontal transfer of pESI *in vivo* can occur during intestinal *Salmonella* infection. To test this hypothesis, C57BL/6 female mice were infected intragastrically with *S*. Infantis 119944 harboring pESI, and their microbiota was subsequently screened for the acquisition of pESI. In the course of 5 months postinfection (p.i.), mouse feces were sampled at different time points and plated onto brucella blood agar plates supplemented with hemin, vitamin K_1_, tetracycline, trimethoprim, and sulfamethoxazole, which were incubated at 37°C under anaerobic conditions. Bacteria colonies that appeared on these plates within 14 days were probed by PCR and dot blot hybridization for the presence of pESI-specific genes. The screening protocol used for the isolation of microbiota transconjugants is illustrated in more detail in Fig. S2 in the supplemental material.

**FIG 6:**
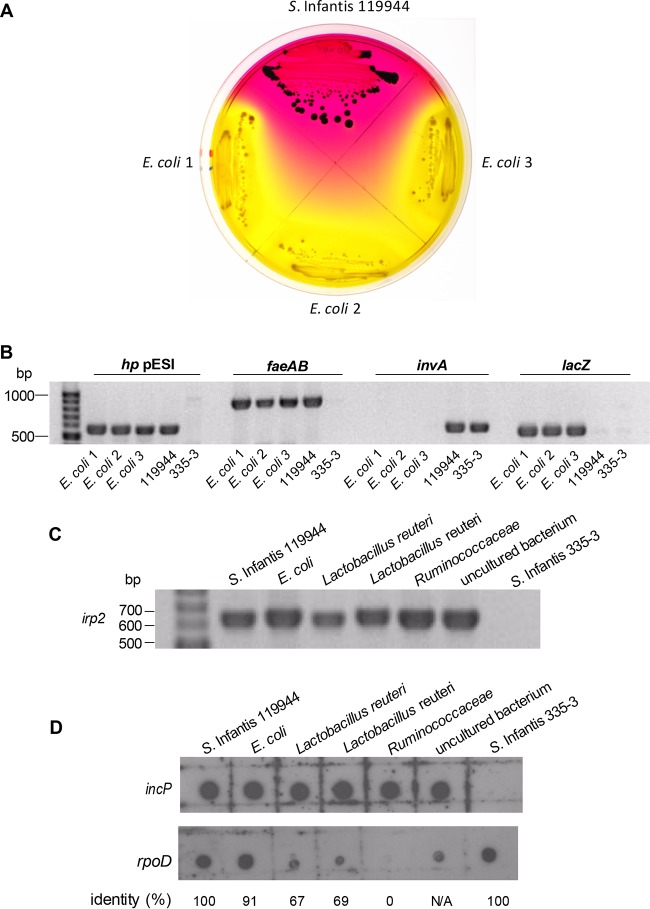
Interspecies transfer of pESI during *S*. Infantis infection. Female C57BL/6 mice were infected with 1.5 × 10^8^ CFU of *S*. Infantis strain 119944 (harboring pESI) and screened for non-*Salmonella* microbiota that have acquired pESI following the infection. (A) The donor (*S*. Infantis 119944) and three mouse-isolate microbiota *E. coli* transconjugants were plated on XLD plates supplemented with tetracycline. (B) To confirm the presence of pESI in the above *E. coli* transconjugants, PCR amplification of pESI-specific genes (*hp* pESI and *faeAB*) and *Salmonella*-specific (*invA*) and *E. coli*-specific (*lacZ*) genes was conducted. *S*. Infantis 119944 and *S*. Infantis 335-3 lacking pESI were used as positive and negative controls, respectively. (C) Five representative microbiota isolates, including *E. coli* (isolate 481-49), *L. reuteri* (isolates 480-44 and 482-46), *Ruminococcaceae* (isolate 482-50), and an unknown bacterium (isolate 481-27) were subjected to PCR using primers from the pESI-specific gene *irp2*. *S*. Infantis 119944 and a pESI-negative *S*. Infantis isolate (335-3) were used as positive and negative controls, respectively. (D) Total DNA (100 ng) that was extracted from the above microbiota isolates was subjected to a dot blot hybridization using a DIG-labeled *incP* pESI backbone probe. A DIG-labeled *rpoD* probe was used as a hybridization control, and the identity (in percentage) between the *rpoD* sequence of *S*. Infantis (used to synthesize the *rpoD* probe) and its homolog in the tested genome is shown at the bottom of the panel.

At 6 days p.i., three independent gut microbiota colonies that were tetracycline, trimethoprim, and sulfamethoxazole resistant were isolated. 16S rRNA gene sequencing showed 100% identity to *E. coli* strain SF-166 (GenBank accession no. CP012633). Plating on selective xylose-lysine-deoxycholate (XLD) plates (Fig. 6A) and PCR amplification of genes specific for pESI (*hp* pESI and *faeAB*), *Salmonella* (*invA*), and *E. coli* (*lacZ*) (Fig. 6B) confirmed the presence of pESI in these mouse-isolate *E. coli* microbiota members.

In addition to these *E. coli* transconjugants and at later time points p.i., 30 Gram-positive bacteria (mostly *Lactobacillus reuteri*) that were tetracycline, sulfamethoxazole, and trimethoprim resistant were isolated from the infected mice (see Table S3 in the supplemental material). PCR amplification of several pESI-specific genes, including *irp*2 (Fig. 6C), *hp* pESI, and *faeAB* (data not shown) as well as dot blot analysis using a pESI-specific probe (*incP*) (Fig. 6D) showed the presence of pESI DNA in these initially isolated colonies, providing evidence for the transfer of pESI into Gram-positive microbiota members*.* Nevertheless, whole-genome sequencing of five *L. reuteri* isolates following two stages of subculturing on tryptic soy blood agar (TSBA) plates failed to detect pESI in their *de novo-*assembled genomes. These results suggest that although pESI may have transiently acquired by these microbiota species, it was probably unstable and incapable of replicating within *L. reuteri.*

### Gut microbiota can transfer pESI to a medically relevant pathogen.

**FIG 7:**
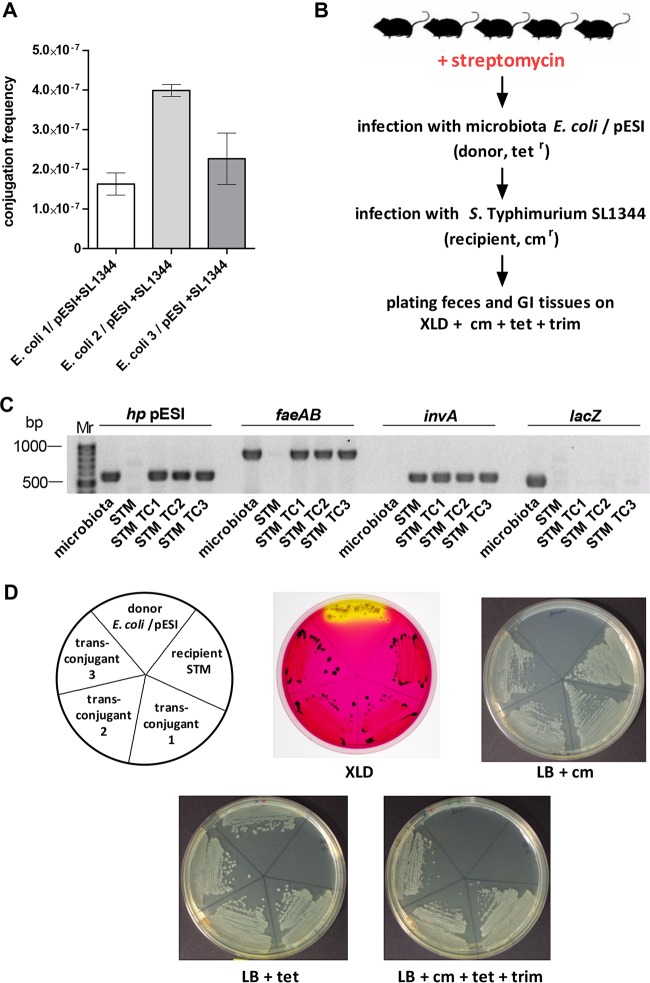
pESI transfer from microbiota to a naive pathogen during infection. (A) Three *E. coli* mouse isolates that have acquired pESI following *S*. Infantis 119944 infection were used as donor strains for conjugation with *S*. Typhimurium SL1344 carrying a chloramphenicol chromosomally carried marker as a recipient strain. pESI conjugation frequency was determined after 16 h at 37°C under microaerobic conditions, by the number of the obtained transconjugants (cm^r^ tet^r^ *S*. Typhimurium) per donor CFU. (B) Experimental workflow used to detect pESI transfer from microbiota to *S*. Typhimurium during infection. Five 7-week-old female C57BL/6 mice were treated with streptomycin 1 day before infection with 1.3 × 10^7^ CFU of mouse isolate *E. coli* harboring pESI (donor). Two days p.i., these mice were reinfected with 1.6 × 10^7^ CFU of *S*. Typhimurium SL1344 (STM) carrying the chloramphenicol marker (recipient). At day 6 p.i., mice were sacrificed and GI tissues were homogenized. Feces and tissue homogenates were plated onto XLD plates supplemented with tetracycline (tet), trimethoprim (trim), and chloramphenicol (cm). (C) To confirm the presence of pESI in three *S*. Typhimurium SL1344 transconjugants (STM TC1-3), PCR amplification of pESI-specific (*hp* pESI and *faeAB*), *Salmonella*-specific (*invA*), and *E. coli*-specific (*lacZ*) genes was conducted. The microbiota *E. coli* strain (donor) and *S*. Typhimurium (recipient) were also included as positive and negative controls, respectively. (D) The *E. coli* microbiota donor, the *S*. Typhimurium (STM) recipient, and the obtained three STM transconjugants were plated on XLD plates and LB plates supplemented with chloramphenicol, tetracycline, and chloramphenicol+tetracycline+trimethoprim.

To further learn if pESI is maintained as an autonomously replicating entity in the *E. coli* microbiota isolates, *in vitro* mating experiments were conducted using the three mouse isolates of *E. coli* as donor strains and *S*. Typhimurium SL1344 carrying a chromosome-encoded chloramphenicol resistance marker as a recipient. pESI transfer from the microbiota *E. coli* was detected at a frequency (2 × 10^−7^ to 4 × 10^−7^) similar to that seen when *S*. Infantis 119944 was used as a donor (Fig. 7A), suggesting that the conjugation system is fully functional and pESI is maintained as an extrachromosomal episome in these microbiota *E. coli* isolates.

Subsequently, we further tested the dissemination of pESI from the microbiota to pathogenic species *in vivo*. Thus, the streptomycin-pretreated mouse model was used, and a group of five C57BL/6 female mice were infected with 1.3 × 10^7^ CFU of mouse-isolate *E. coli* harboring pESI (transconjugant 1). None of these infected mice showed any signs of disease following this infection. Two days post-*E. coli* infection, the mice were reinfected with *S*. Typhimurium SL1344. Mouse feces and GI tract organs were homogenized and plated on XLD plates supplemented with tetracycline, trimethoprim, and chloramphenicol to select for *S*. Typhimurium pESI transconjugants as schematically illustrated in Fig. 7B. At day 5 p.i., one *S*. Typhimurium transconjugant was isolated from the feces, and at day 6 p.i., after the mice were sacrificed, two additional *S*. Typhimurium transconjugants were isolated from the jejunum and colon of the same infected mouse. Confirmation of pESI acquisition by *S*. Typhimurium SL1344 was done by PCR amplification of pESI-specific (*hp* pESI and *faeAB*), *Salmonella* (*invA*), and *E. coli* (*lacZ*) genes (Fig. 7C) and by plating the isolates on XLD and selective LB plates (Fig. 7D). These results demonstrated the ability of the gut microbiota (commensal *E. coli* in this case) to transfer the virulence and resistance megaplasmid pESI to a different pathogenic species in the context of infection.

## DISCUSSION

Different derivatives of pESI were recently found to be associated with emergent strains of *S*. Infantis (7, 9). Illuminating the ecology, regulation, and transmission properties of pESI will add to the current understanding of the global *S*. Infantis emergence. Screening of recent *S*. Infantis isolates demonstrated the fixation of pESI in the domestic *S*. Infantis population. This observation and the single copy number of pESI are consistent with the finding that at least *in vitro*, pESI does not impose a significant metabolic burden on its bacterial host. A low- or even single-copy-number plasmid is somewhat expected, as other large conjugative plasmids were also shown to be maintained in a very low copy number (25).

Because they are energy-consuming processes and because conjugative pili are often utilized by bacteriophage as receptors for bacterial infection (26), pilus assembly and conjugation are usually tightly regulated, ensuring the expression of this system in a spatially and timely appropriate manner (27). A fundamental aspect of HGT is the assimilation of the newly acquired genes into the preexisting regulatory setup of the new carrier (28, 29). Insights into this mechanism were obtained by the identification of two regulators controlling *pil* gene expression and pESI conjugation. The gene coding for TraB is carried by pESI, and its homolog in the R64 plasmid was previously suggested to encode a putative positive regulator of T4SS genes (30). Here we established that TraB is a transcriptional regulator of the *pil* genes in pESI. The second identified regulator is FNR, a global oxygen-responsive transcriptional regulator required for the switch from aerobic to anaerobic metabolism (31). In *S*. Typhimurium, FNR was previously shown to regulate more than 300 genes involving in aerobic metabolism, nitric oxide detoxification, anaerobic carbon, and ethanolamine utilization, as well as the transcription of numerous *Salmonella* pathogenicity island 1 flagellar chemotaxis and virulence genes (32). Under microaerobic conditions, the absence of FNR resulted in a downregulation of *pilV*, *pilS*, and *pilT* transcription and lower pESI conjugation, demonstrating the integration of the pilus gene expression with the global metabolic regulatory setup of *Salmonella*.

pESI conjugation was further shown to be regulated in response to specific environmental cues, including oxidative stress, physiological temperature, and moderate osmolarity. It was also found that under moderately acidic pH and physiological temperatures, transcription of the conjugation system (*pilV*) was increased. Temperature-dependent conjugation has been previously reported for the transfer-thermosensitive IncHI1 plasmids, such as R27, as its conjugation frequency is optimal at low temperature (25 to 30°C) and declines when temperature increases (33). In contrast to the IncHI1 plasmids, pESI conjugation and *pil* gene expression are minimal at 27°C while increasing at 37 to 41°C. This suggests that different temperatures are used by plasmids as an environmental signal to control their transfer, in a way that is likely to be associated with their distinct ecological niches or lifestyles (e.g., environmental or cold-blooded versus warm-blooded hosts).

As opposed to the elevated transfer under microaerobiosis, physiological temperature, and moderate osmolarity, pESI conjugation and *pilV* and *pilT* transcription were found to be suppressed in the presence of ≥1% bile salts. Similar results have been previously reported for pSLT conjugation (34). Bile salts are secreted into the small intestine, reaching a concentration range of 0.2% to 2%; however, 98% of the bile is being absorbed in the terminal ileum by an active transport mechanism that is highly efficient, resulting in a much lower concentration of bile in the large intestine (35). Taken together, the *in vitro* conjugation results indicate that pESI transfer is prompted under environmental conditions found in the gut of warm-blooded animals and, possibly, the midcolon, characterized by high osmolality (18), 1.4% oxygen (36), a pH of 5 to 7 (37), and low bile salt concentration (37). This is highly reasonable since the animal gut is known to be occupied by a wealth of microorganisms of at least 1,000 different species (38) that can reach to 10^11^ to 10^12^ cells/ml (37), providing abundant conjugation opportunities. Thus, hosting the largest microbial community in the human body, the GI tract has been long suggested as “hot spot” for HGT between microbes (39–41).

As a proof of this concept, we utilized the mouse infection model and demonstrated interspecies transfer of pESI from *S*. Infantis to *E. coli*—members of the mouse gut microbiota. Furthermore, different experimental approaches provided evidence for initial pESI transfer into Gram-positive microbiota as well. However, whole-genome sequencing following two stages of subculturing in laboratory medium failed to detect pESI in the assembled genomes, suggesting that pESI cannot persist in these Gram-positive hosts. These results add to the previous few reports that were able to show conjugative transfer of a naturally occurring plasmid between Gram-negative and Gram-positive bacteria (42–44). An interesting overrepresentation of pESI DNA was found particularly in *Lactobacillus* spp., possibly due to their hydrogen peroxide production (45, 46), shown here to further induce *pilV* transcription as well as pESI conjugation.

To demonstrate a complete turnover of pESI transfer, we further used the isolated pESI-acquired microbiota to infect streptomycin-pretreated mice followed by infection with *S*. Typhimurium. In this experiment, we were able to demonstrate secondary transfer of pESI from the commensal *E. coli* to a naive pathogen during infection.

Horizontal transfer of virulence and resistance genes from a pathogen to the microbiota and then further to a different pathogen *in vivo* is both intriguing and worrying. Several recent studies reported anecdotal HGT from pathogens to the microbiota in the human GI tract (47–49); however, the role of the human or livestock microbiota in transfer of virulence genes is still underappreciated. A few years ago, Salyers and colleagues proposed the resistance gene reservoir hypothesis, suggesting that intestinal bacteria serve as a reservoir for antibiotic resistance genes that can be transferred to other intestinal colonizers or transient bacteria (50). Our results provide experimental support for this theory and demonstrate the potential role of the gut microbiota as a mediator for genetic flux of virulence and resistance elements, in the physiological context of an intestinal infection.

It is noteworthy that several inherent limitations and caveats of the experimental setup used are likely to offer only a narrow window into the actual HGT events that occurred using this model system. Although brucella blood agar medium is used for the isolation of a wide array of anaerobes from clinical specimens (51), other gut microbiota species with different growth requirements or that are viable but nonculturable could not be isolated and analyzed by the described workflow. Thus, it is highly likely that we were incapable to detect many or even most of the actual conjugation events that happened *in vivo*. Yet, we were still able to demonstrate HGT of pESI from a pathogen into the mouse microbiota and back to a different *Salmonella* serovar. Even if this event happens *de facto* at a very low frequency (~10^−7^), it may still be a significant mechanism in the evolution of new pathogens and emergence of resistant strains.

In summary, we gained evolutionary insights into the modular formation of a clinically relevant megaplasmid and demonstrated its current fixation in the domestic *S*. Infantis population. We showed that the expression of the pilus genes and pESI conjugation are controlled both by a pESI-encoded (TraB) and global (FNR) regulators and induced in response to specific environmental conditions, typical to the midcolon. Using the mouse model, we have established interspecies transfer of pESI from *S*. Infantis to members of the microbiota and back to a new medically relevant pathogen. These results illuminate a potential role of the microbiota community as a bridge for the transfer of genes between pathogenic and commensal bacteria, exhibiting that bugs may not have sex very often, but when they do, it can be impactful.

## MATERIALS AND METHODS

### Bacterial strains, media, and growth conditions.

The bacterial strains utilized in this study are listed in Table S1 in the supplemental material. *S*. Infantis isolates were obtained from the Israeli national *Salmonella* reference center after serotyping according to the White-Kauffmann Le-Minor scheme. *E. coli* K-12 ORN172 (km^r^) was a generous gift from the Paul Orndorff Laboratory. Bacterial cultures were routinely grown in rich Luria-Bertani (LB) broth (Lennox) or in N-minimal medium. Microbiota was cultivated on brucella blood agar plates supplemented with hemin and vitamin K_1_ (BD Difco) or tryptic soy blood agar (TSBA [Hylabs, Israel]). More details are provided in Text S1 in the supplemental material.

### Fitness assay.

Growth competition experiments were performed between an *S*. Infantis 335-3 strain carrying pESI (335-3-pESI) against a plasmidless *S*. Infantis 335-3 strain. One milliliter of overnight cultures was centrifuged, resuspended in 1 ml of N-minimal medium, and normalized to an optical density at 600 nm (OD_600_) of 5. From the OD-normalized cultures, 8 µl was inoculated into 4 ml of LB or N-minimal medium with no antibiotics and grown with shaking (250 rpm) at 27°C. After 24 h, the mixed culture was diluted and plated onto LB plates and onto LB plates supplemented with 20 µg/ml tetracycline for numeration of pESI-containing CFU. The competitive index was calculated as (335-3-pESI/335-3)_output_/(335-3-pESI/335-3)_input_.

### S1 nuclease digestion and PFGE analysis.

Plasmid size was determined by S1 nuclease digestion followed by PFGE. Cells of *S*. Infantis isolates 119944 and 120100, which were grown on nutrient agar plates overnight, were suspended in cell suspension buffer (100 mM Tris, 100 mM EDTA [pH 8.0]) to an OD_600_ of 1.3 to 1.4 and used to cast DNA extraction agarose plugs. DNA plugs were incubated with 1.7 U of S1 nuclease (Sigma-Aldrich) in a restriction buffer (0.2 M Nacl, 2 mM ZnSO_4_, 60 mM acetic acid [pH 4.6]) for 1 h at 37°C. Digestion was stopped by incubation of the plugs with 200 µl of ES buffer (1% sodium lauroylsarcosine, 0.5 M EDTA) on ice. Digested DNA was then separated by PFGE according to the PulseNet protocol (52).

### Next-generation sequencing and bioinformatics.

Whole-genome sequencing was performed at the Technion Genome Center (Haifa, Israel) using the MiSeq platform and 500 or 600 cycles of Illumina’s paired-end chemistry to determine the genome sequence of *S*. Infantis 120100 and five *L. reuteri* microbiota isolates to a draft level. The obtained reads provided at least 300-fold coverage and were subjected to *de novo* assembly using the CLC Genomics workbench 6 package (CLC-bio, Denmark).

### Mating experiments.

pESI transfer by conjugation was performed using *S*. Infantis strain 119944 or the mouse isolate *E. coli* strain (transconjugant 1) as the donor and *E. coli* ORN172 (kanamycin resistant), J5-3 (rifampin resistant), or *S*. Typhimurium SL1344 (chloramphenicol resistant) as the recipient strain on LB agar plates for 16 h at 37°C unless otherwise stated. Aerobic conjugation was tested after both strains were grown in LB for 16 h with aeration, and 1 ml from each strain was harvested by centrifugation and resuspended in 100 µl of LB medium. Equal amounts (10 µl; ~2 × 10^9^ CFU) from each culture were mixed and placed onto LB agar plates supplemented with different concentrations of NaCl, bile, or hydrogen peroxide. Anaerobic conditions were achieved using the GasPak EZ anaerobe container system (BD Difco). A sealed gas jar with a CampyGen 2.5-liter sachet (Thermo Scientific) was used to create microaerobic conditions (6.2% to 13.2% O_2_). The conjugation mixture was scraped from the plate and resuspended in LB broth, and serial dilutions were plated on LB agar plates supplemented with tetracycline (to select for pESI) and kanamycin, chloramphenicol, or rifampin to select for the appropriate recipient strain.

### Evaluation of pESI copy number.

Total DNA was extracted from an overnight culture of *S*. Infantis strain 335-3 carrying pESI, using the GeneElute bacterial genomic DNA kit (Sigma-Aldrich). Ten-fold serial dilutions of the genomic DNA ranging from undiluted to 10^−5^ (1,200 to 0.012 ng) were used to prepare standard curves for the pESI-carried gene *pilV* and the chromosome-carried (single-copy) gene *rpoD*, which was used as a reference. Threshold cycle (*C_T_*) values for each dilution were measured in triplicates on a StepOnePlus real-time PCR system (Applied Biosystems) using FastStart Universal SYBR green master (ROX) mix (Roche Applied Science). The percentage of PCR amplification efficiency (*E*) was calculated from the slope of the standard curve using the following formula: *E* = (10^−1/slope^ − 1) × 100.

### Reverse transcription–real-time PCR experiments.

RNA was extracted from *Salmonella* cultures grown under different conditions using the Qiagen RNAprotect bacterial reagent and the RNeasy minikit (Qiagen) according to the manufacturer’s instructions, including an on-column DNase digest. Real-time PCRs (RT-PCRs) were performed as previously described (53). The 16S rRNA gene was used as the endogenous normalization control. Fold differences in gene expression were calculated as 2^−ΔΔ*CT*^.

### Molecular biology and cloning.

The primers used in this study are listed in Table S2 in the supplemental material. Oligonucleotides were purchased from IDT, and PCR was carried out using ReddyMix PCR (Thermo Scientific) or with PfuUltra II fusion HS DNA polymerase (Stratagene). In-frame deletion *S*. Infantis mutants were constructed as described in reference 54 using the lambda red recombinase system and a three-step PCR method to produce an amplimer containing the antibiotic resistance gene. For *fnr* and *traB* cloning, the intact sequence of the two genes was PCR amplified, using *S*. Infantis 119944 as a template. The obtained DNA fragments were digested with XbaI and XhoI and ligated into pWSK29.

### Dot blotting hybridization.

Genomic DNA was extracted using the GeneElute bacterial genomic DNA kit (Sigma-Aldrich), and 100 ng from each genome was spotted onto Hybond N+ membrane (GE Healthcare, Amersham), UV cross-linked, and hybridized with specific pESI backbone *incP* and *rpoD* digoxigenin (DIG)-labeled probes. The probes were labeled using the PCR DIG probe synthesis kit (Roche). The blots were hybridized overnight at 45°C in a DIG-Easy hybridization solution and washed twice with 2× SSC (1× SSC is 0.15 M NaCl plus 0.015 mM sodium citrate)–0.1% SDS at room temperature, followed by a 15-min wash with 0.1× SSC–0.1% SDS at 68°C. The detection was done using anti-digoxigenin–alkaline phosphatase (AP), Fab fragments, and CDP-Star substrate (Roche).

### pESI transfer in the mouse infection model.

The experimental setup of the mouse infection model is illustrated in detail in Fig. S2 in the supplemental material. The mouse experiments were approved and carried out according to the Israeli national animal care guidelines and the institutional ethics committee of the Sheba Medical Center (approval no. 601/10). Briefly, two groups of mice (streptomycin treated and untreated) were orally infected with 1.5 × 10^8^ CFU of *S*. Infantis 119944. Mouse feces were collected over 159 days with 7-day intervals between samplings. At each time point, mouse pellets were plated on brucella blood agar plates supplemented with hemin and vitamin K_1_ (BD Difco), under the selection of tetracycline, sulfamethoxazole, and trimethoprim, used to select for microbiota that have acquired pESI. After 14 days of incubation anaerobically, non-*Salmonella* isolates that were resistant to the above three antibiotics were screened by PCR for 3 pESI backbone genes (*incP*, *hp*, and *faeAB*) and *ssaR* (*Salmonella*-specific gene). Confirmed non-*Salmonella* isolates (*incP*, *hp*, and *faeAB* positive and *ssaR* negative) were considered pESI positive and subjected to a Gram staining and 16S rRNA sequencing to determine their phylogenetic classification. Similarly, streptomycin-treated mice were infected with 1.3 × 10^7^ CFU of mouse-isolate *E. coli* harboring pESI. Two days p.i., these mice were reinfected with 1.6 × 10^7^ CFU of *S*. Typhimurium SL1344 carrying the chloramphenicol chromosomal marker. Feces were sampled every other day. Mice were sacrificed at day 6 p.i., and GI tract organs were homogenized in saline and plated onto XLD plates supplemented with tetracycline, trimethoprim, and chloramphenicol.

### Statistics.

The *Z* score test for two population proportions with a two-tailed hypothesis was used to compare the prevalence of pESI. A D’Agostino and Pearson omnibus normality test was used to determine Gaussian distributions of pESI conjugation and *pil* gene expression data. A two-tailed one-sample *t* test was used to determine differences between two groups, and analysis of variance (ANOVA) with Dunnett’s or Tukey’s multiple comparison tests was used to determine differences between multiple data sets. A *P* value smaller than 0.05 was considered statistically significant.

### Accession number(s).

Sequences determined in the whole-genome shotgun projects have been deposited in DDBJ/EMBL/GenBank under accession no. LMXO00000000 (*S*. Infantis 120100), MBLQ00000000 (*L. reuteri* 480_44), MBLR00000000 (*L. reuteri* 482_46), MBLS00000000 (*L. reuteri* 482_54), MBLT00000000 (*L. reuteri* 484_32), and MBLU00000000 (*L. reuteri* 484_39).

## SUPPLEMENTAL MATERIAL

Text S1 Supplemental materials and methods. Download Text S1, DOCX file, 0.02 MB

Figure S1  Complete fixation of pESI in the *S*. Infantis population. The presence of pESI was examined in different *S*. Infantis isolates from clinical (*n* = 16), food (*n* = 8), and poultry (*n* = 24) sources using pESI backbone-specific primers. *S*. Infantis isolate 119944 harboring pESI and the pESI-negative isolate 335-3 were used as a positive control and negative control, respectively. Download Figure S1, PDF file, 0.1 MB

Figure S2  Screening of pESI acquisition by mouse microbiota members. Eight- to 10-week-old female C57BL/6 mice were purchased from Harlan Laboratories and housed at the Sheba Medical Center animal facility under specific-pathogen-free conditions. Experiments in this study were approved and carried out according to the national animal care guidelines and the institutional ethics committee of the Sheba Medical Center (approval no. 601/10). Before the infection, no bacterial growth was observed from feces that were plated onto brucella blood agar plates supplemented with hemin, vitamin K_1_, tetracycline, trimethoprim, and sulfamethoxazole. Mice were infected with 1.5 × 10^8^ CFU of *S*. Infantis strain 119944 carrying pESI in 200 µl HEPES buffer. Streptomycin (20 mg per mouse) was given by oral gavage 24 h prior to infection to one group (*n* = 4) of mice. Following the infection, feces were collected at 7-day intervals, homogenized in 700 µl saline, and plated on XLD plates supplemented with tetracycline for *Salmonella* enumeration and onto brucella blood agar plates supplemented with hemin, vitamin K_1_, tetracycline, trimethoprim, and sulfamethoxazole for microbiota transconjugant isolation. The brucella plates were immediately incubated under anaerobic conditions, inside the GasPak EZ jar, at 37°C for 14 days. Tetracycline-, trimethoprim-, and sulfamethoxazole-resistant colonies were picked up from the brucella plates and restreaked on XLD plates for *Salmonella* detection and on new brucella selective plates (which were incubated again for 14 days under anaerobic conditions). Colonies that did not grow on XLD (or appear as non-*Salmonella*) and grew on selective brucella plates were screened by PCR for pESI backbone genes (*hp* and *faeAB*) and for the *Salmonella*-specific gene (*ssaR*). Non-*Salmonella* colonies that were positive for pESI were subjected to 16S rRNA sequencing and Gram staining for taxonomic classification. To confirm the presence of pESI, representative isolates were subjected to PCR with the *irp2* gene (another pESI-specific gene) and dot blotted with *incP* and *ropD* DIG-labeled probes as shown in Fig. 6C and D, respectively. Download Figure S2, PDF file, 0.4 MB

Table S1  Bacterial strains utilized in this study. Table S1, DOCX file, 0.03 MB

Table S2  Primers used in this study. Table S2, DOCX file, 0.02 MB

Table S3  Mouse microbiota isolates found positive for pESI. Table S3, DOCX file, 0.02 MB
