# Supplementary material for: Horizontal Transfer of the Salmonella enterica Serovar Infantis Resistance and Virulence Plasmid pESI to the Gut Microbiota of Warm-Blooded Hosts
Source: mBio. 2016 Sep 6;7(5):e01395-16. doi: 10.1128/mBio.01395-16 (PMC5013300; doi:10.1128/mBio.01395-16)
Supplement: Text S1 — Supplemental materials and methods. Download [file mbo004162973s1.docx]

**Supplementary Information**

**Materials and Methods**

**Media and growth conditions.** *Salmonella* and *E. coli* cultures were routinely maintained in Luria-Bertani (LB; BD Difco) liquid broth or in N-minimal media medium containing 80 mM MES (pH 5.8) or 100 mM Tris-HCl (pH 7.0), 5 mM KCl, 7.5 mM (NH_4_)SO_4_, 0.5 mM K_2_SO_4_, 337 μM K_2_HPO_4_/KH_2_PO_4_, 20 mM MgCl_2_, 38 mM glycerol, and 0.1% Casamino acids ([1](#_ENREF_1)) and plated onto LB or xylose lysine deoxycholate (XLD; BD Difco) agar plates. Microbiota from mice feces were cultivated on *Brucella* blood agar plates (BD Difco) containing 10 g/l pancreatic digest of casein; 10 g/l peptic digest of animal tissue; 2 g/l yeast extract, 1g/l glucose; 5 g/l NaCl; 0.1 g/l NaHSO_3_; 0.005 g/l Hemin; 0.01 g/l Vitamin K1; 5% Sheep Blood, defibrinated and 15 g/l agar. This is a highly nutritious medium that uses for the isolation and cultivation of anaerobes from clinical specimens ([2](#_ENREF_2)). For microbiota isolation the *Brucella* blood plates were incubated under anaerobic conditions using the GasPak EZ anaerobe container system with indicator (BD Difco) at 37° C for 14 days. *Lactobacillus reuteri* isolates were grown on tryptic soy blood agar (TSBA) plates with 5% defibrinated sheep blood (Hylabs, Israel). When appropriate, antibiotics were added to the medium as follows: tetracycline (20 μg/ml), nitrofurantoin (64 μg/ml), trimethoprim (50 μg/ml), nalidixic acid (20 μg/ml), sulfamethoxazole (50 μg/ml), kanamycin (50 μg/ml), ampicillin (100 μg/ml) and chloramphenicol (10-25 μg/ml). Crude ox bile extract (Sigma-Aldrich) was added to LB broth or LB agar as indicated.

**Supplementary Figures legends**

**Figure S1. The complete fixation of pESI in the *S*. Infantis population.** The presence of pESI was examined in 49 different *S*. Infantis isolates from clinical (n=16), food (n=8) and poultry (n=24) sources using pESI backbone specific primers. *S*. Infantis isolate 119944 harboring the pESI and the pESI-negative, isolate 335-3 were used as a positive and a negative controls, respectively.

**Figure S2.** **Screening of pESI acquisition by mouse microbiota members.** Eight to ten-weeks-old female C57/BL6 mice were purchased from Harlan laboratories and housed at the Sheba Medical Center animal facility under specific pathogen free conditions. Experiments in this study were approved and carried out according to the national animal care guidelines and the institutional ethics committee of the Sheba Medical Center (approval No 601/10). Before the infection, no bacterial growth was observed from feces that were plated onto *Brucella* blood agar plates supplemented with hemin, vitamin K1, tetracycline, trimethoprim and sulfamethoxazole. Mice were infected with 1.5×10^8^ *S*. Infantis strain 119944 carrying pESI in 100 μl HEPES buffer. Streptomycin (20 mg per mouse) was given by oral gavage 24 h prior to infection to one group (N=4) of mice. Following the infection, feces were collected at 7 days intervals, homogenized in 700 µl saline and plated on XLD supplemented with tetracycline for *Salmonella* enumeration and onto *Brucella* blood agar plates supplemented with hemin, vitamin K1, tetracycline, trimethoprim and sulfamethoxazole for microbiota transconjugants isolation. The *Brucella* plates were immediately incubated under anaerobic conditions, inside the GasPak EZ jar, at 37°C for 14 days. Tetracycline, trimethoprim and sulfamethoxazole-resistant colonies were picked up from the *Brucella* plates and restreaked on XLD plates for *Salmonella* detection and on new *Brucella* selective plates (that were incubated again for 14 days under anaerobic conditions). Colonies that did not grow on XLD (or appear as non-*Salmonella*), and grew on selective *Brucella* plates were screened by PCR for pESI backbone genes (*hp* and *faeAB*) and for *Salmonella* specific gene (*ssaR* or *invA*). Non-*Salmonella* colonies that were positive for pESI were subjected to 16S rRNA sequencing and Gram-staining for taxonomic classification. To confirm pESI presence, representative isolates were subjected to PCR with *irp2* gene (another pESI specific gene) and dot blot with *incP* and *ropD* DIG-labeled probes as shown in Fig. 6C and 6D, respectively.

**References**

1. **Coombes BK, Brown NF, Valdez Y, Brumell JH, Finlay BB.** 2004. Expression and secretion of *Salmonella* pathogenicity island-2 virulence genes in response to acidification exhibit differential requirements of a functional type III secretion apparatus and SsaL. J Biol Chem **279:**49804-49815.

2. **Summanen P BEJ, Citron D M, Strong C A, Wexler H M, Finegold S M.** 1993. Wadsworth anaerobic bacteriology manual, 5th ed. Star Publishing Co.
