# Supplementary figures and images for: Horizontal Transfer of the Salmonella enterica Serovar Infantis Resistance and Virulence Plasmid pESI to the Gut Microbiota of Warm-Blooded Hosts
Source: mBio. 2016 Sep 6;7(5):e01395-16. doi: 10.1128/mBio.01395-16 (PMC5013300; doi:10.1128/mBio.01395-16)

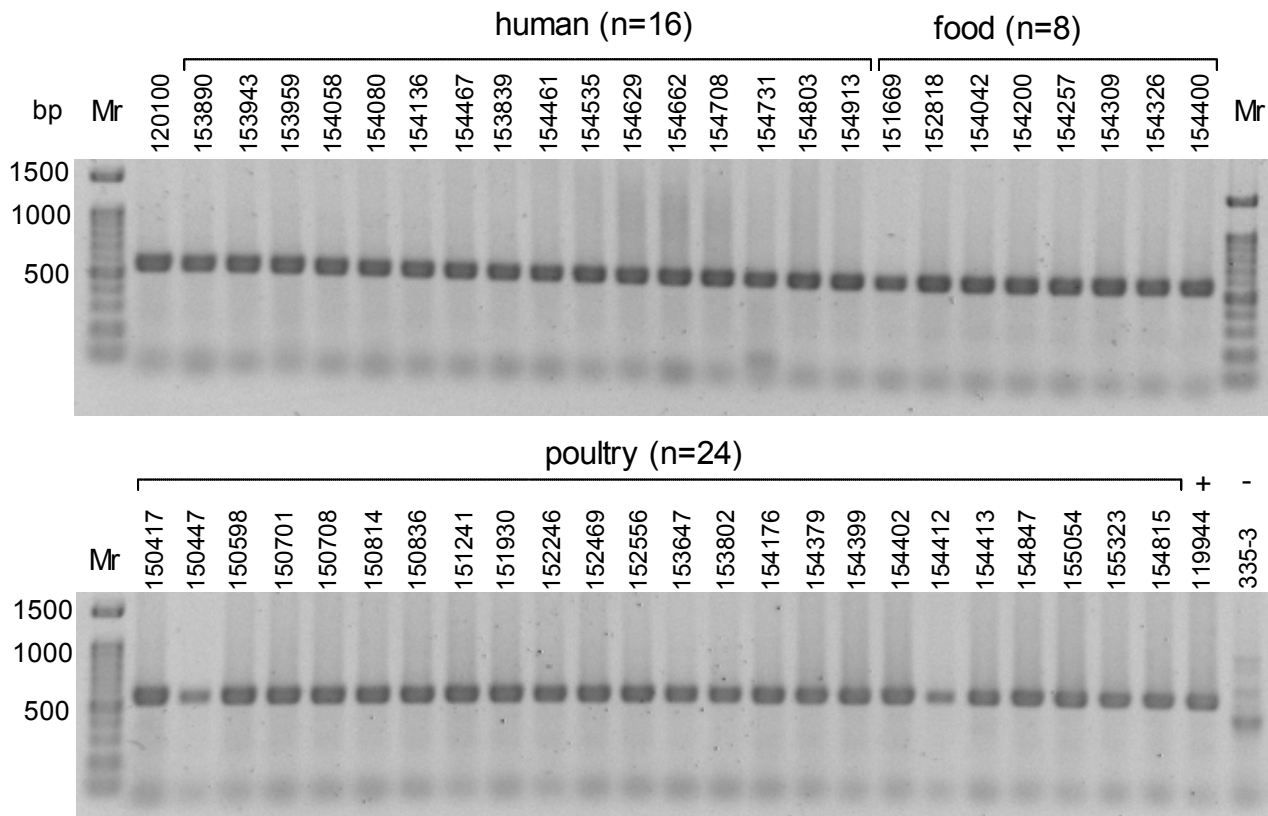

**Fig. S1**

Supplement: Figure S1 — Complete fixation of pESI in the S. Infantis population. The presence of pESI was examined in different S. Infantis isolates from clinical (n = 16), food (n = 8), and poultry (n = 24) sources using pESI backbone-specific primers. S. Infantis isolate 119944 harboring pESI and the pESI-negative isolate 335-3 were used as a positive control and negative control, respectively. Download [file mbo004162973sf1.pdf]
