# Supplementary material for: Horizontal Transfer of the Salmonella enterica Serovar Infantis Resistance and Virulence Plasmid pESI to the Gut Microbiota of Warm-Blooded Hosts
Source: mBio. 2016 Sep 6;7(5):e01395-16. doi: 10.1128/mBio.01395-16 (PMC5013300; doi:10.1128/mBio.01395-16)
Supplement: Figure S2 — Screening of pESI acquisition by mouse microbiota members. Eight- to 10-week-old female C57BL/6 mice were purchased from Harlan Laboratories and housed at the Sheba Medical Center animal facility under specific-pathogen-free conditions. Experiments in this study were approved and carried out according to the national animal care guidelines and the institutional ethics committee of the Sheba Medical Center (approval no. 601/10). Before the infection, no bacterial growth was observed from feces that were plated onto brucella blood agar plates supplemented with hemin, vitamin K1, tetracycline, trimethoprim, and sulfamethoxazole. Mice were infected with 1.5 × 10^8^ CFU of S. Infantis strain 119944 carrying pESI in 200 µl HEPES buffer. Streptomycin (20 mg per mouse) was given by oral gavage 24 h prior to infection to one group (n = 4) of mice. Following the infection, feces were collected at 7-day intervals, homogenized in 700 µl saline, and plated on XLD plates supplemented with tetracycline for Salmonella enumeration and onto brucella blood agar plates supplemented with hemin, vitamin K1, tetracycline, trimethoprim, and sulfamethoxazole for microbiota transconjugant isolation. The brucella plates were immediately incubated under anaerobic conditions, inside the GasPak EZ jar, at 37°C for 14 days. Tetracycline-, trimethoprim-, and sulfamethoxazole-resistant colonies were picked up from the brucella plates and restreaked on XLD plates for Salmonella detection and on new brucella selective plates (which were incubated again for 14 days under anaerobic conditions). Colonies that did not grow on XLD (or appear as non-Salmonella) and grew on selective brucella plates were screened by PCR for pESI backbone genes (hp and faeAB) and for the Salmonella-specific gene (ssaR). Non-Salmonella colonies that were positive for pESI were subjected to 16S rRNA sequencing and Gram staining for taxonomic classification. To confirm the presence of pESI, representative isolates were subjected [file mbo004162973sf2.pdf]

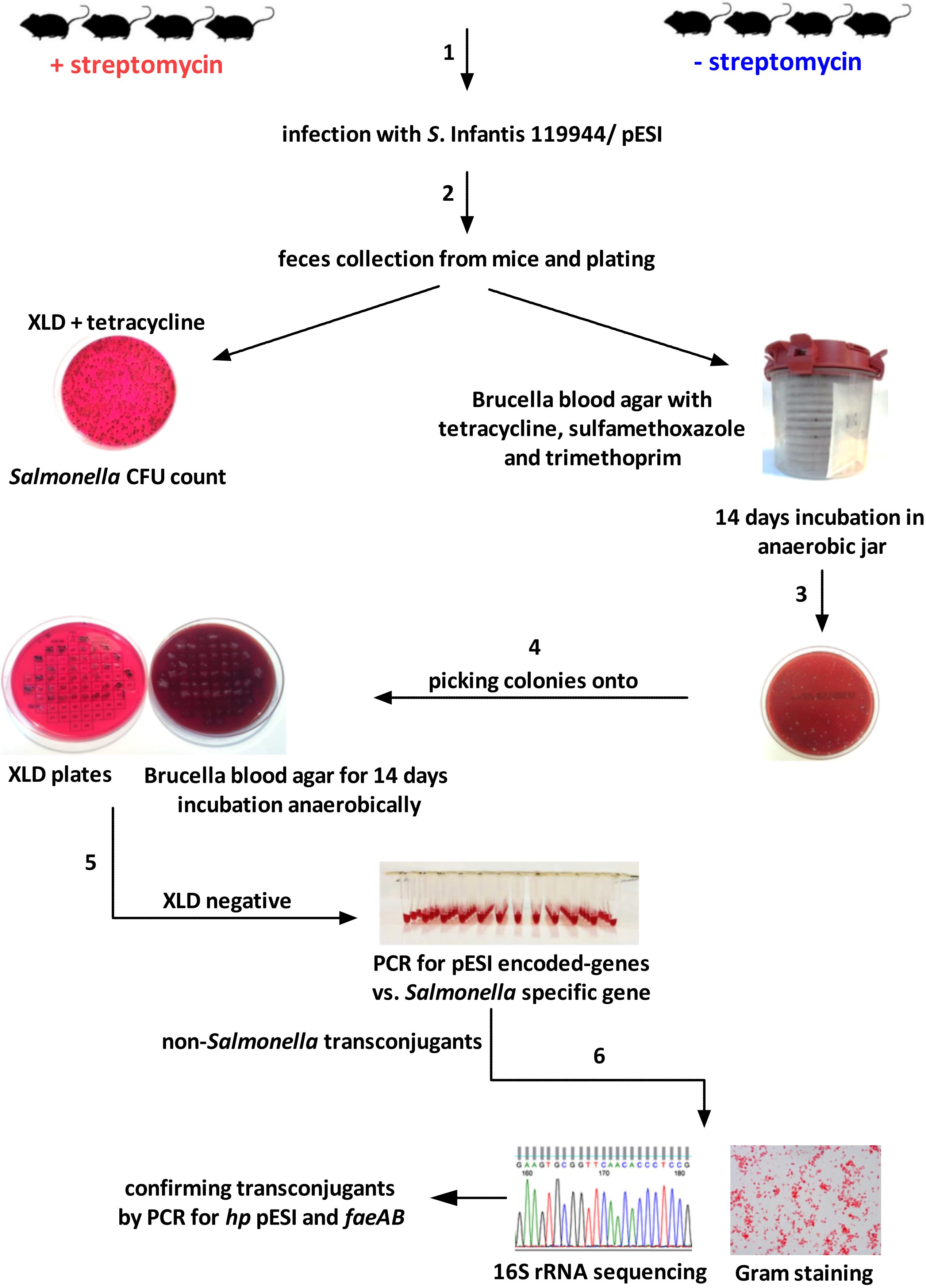
+ streptomycin
- streptomycin
1
infection with *S.* Infantis 119944/ pESI
2
feces collection from mice and plating
XLD + tetracycline
Salmonella CFU count
Brucella blood agar with
tetracycline, sulfamethoxazole
and trimethoprim
14 days incubation in
anaerobic jar
3
4
picking colonies onto
XLD plates
Brucella blood agar for 14 days
incubation anaerobically
5
XLD negative
PCR for pESI encoded-genes
vs. *Salmonella* specific gene
non-*Salmonella* transconjugants
6
confirming transconjugants
by PCR for *hp* pESI and *faeAB*
16S rRNA sequencing
Gram staining
