# Supplementary material for: Horizontal Transfer of the Salmonella enterica Serovar Infantis Resistance and Virulence Plasmid pESI to the Gut Microbiota of Warm-Blooded Hosts
Source: mBio. 2016 Sep 6;7(5):e01395-16. doi: 10.1128/mBio.01395-16 (PMC5013300; doi:10.1128/mBio.01395-16)
Supplement: Table S1 — Bacterial strains utilized in this study. [file mbo004162973st1.docx]

**Table S1. Bacterial strains utilized in this study.**

| **Strain** | **Description** | **Source or Reference** |
| --- | --- | --- |
| *S*. Infantis 119944 | 2008 clinical isolate. Sequenced strain | ([1](#_ENREF_1)) |
| *S*. Infantis 335-3 | 1970 isolate. Sequenced strain | ([1](#_ENREF_1)) |
| *S*. Infantis 335-3/pESI | 335-3 containing the pESI plasmid | ([1](#_ENREF_1)) |
| *E. coli* ORN172 | *thr-l leuB thi-1 ∆(argF-lac)U169 xyl-7 ara-13 mtl-2 gal-6 rpsL tonA2 supE44 ∆(fimBEACDFGH)::kan pilG1* | ([2](#_ENREF_2)) |
| *E. coli* J5-3 | Rifampicin resistant | ([3](#_ENREF_3)) |
| *S*. Typhimurium SL1344 cm^r^ (NB24) | marked strain with Cm cassette at *ushA* gene (*ushA*::res-cat-res) | ([4](#_ENREF_4)) |
| *S*. Infantis 120100 | 2008 food isolate | NSRC |
| 153890 | 2014 clinical isolate | NSRC |
| 153943 | 2014 clinical isolate | NSRC |
| 153959 | 2014 clinical isolate | NSRC |
| 154058 | 2014 clinical isolate | NSRC |
| 154080 | 2014 clinical isolate | NSRC |
| 154136 | 2014 clinical isolate | NSRC |
| 154467 | 2014 clinical isolate | NSRC |
| 153839 | 2014 clinical isolate | NSRC |
| 154461 | 2014 clinical isolate | NSRC |
| 154535 | 2014 clinical isolate | NSRC |
| 154629 | 2014 clinical isolate | NSRC |
| 154662 | 2014 clinical isolate | NSRC |
| 154708 | 2014 clinical isolate | NSRC |
| 154731 | 2014 clinical isolate | NSRC |
| 154803 | 2014 clinical isolate | NSRC |
| 154913 | 2014 clinical isolate | NSRC |
| 150417 | 2014 poultry isolate | NSRC |
| 150447 | 2014 poultry isolate | NSRC |
| 150598 | 2014 poultry isolate | NSRC |
| 150701 | 2014 poultry isolate | NSRC |
| 150708 | 2014 poultry isolate | NSRC |
| 150814 | 2014 poultry isolate | NSRC |
| 150836 | 2014 poultry isolate | NSRC |
| 151241 | 2014 poultry isolate | NSRC |
| 151930 | 2014 poultry isolate | NSRC |
| 152246 | 2014 poultry isolate | NSRC |
| 152469 | 2014 poultry isolate | NSRC |
| 152556 | 2014 poultry isolate | NSRC |
| 153647 | 2014 poultry isolate | NSRC |
| 153802 | 2014 poultry isolate | NSRC |
| 154176 | 2014 poultry isolate | NSRC |
| 154379 | 2014 poultry isolate | NSRC |
| 154399 | 2014 poultry isolate | NSRC |
| 154402 | 2014 poultry isolate | NSRC |
| 154412 | 2014 poultry isolate | NSRC |
| 154413 | 2014 poultry isolate | NSRC |
| 154847 | 2014 poultry isolate | NSRC |
| 155054 | 2014 poultry isolate | NSRC |
| 155323 | 2014 poultry isolate | NSRC |
| 154815 | 2014 poultry isolate | NSRC |
| 151669 | 2014 food isolate | NSRC |
| 152818 | 2014 food isolate | NSRC |
| 154042 | 2014 food isolate | NSRC |
| 154200 | 2014 food isolate | NSRC |
| 154257 | 2014 food isolate | NSRC |
| 154309 | 2014 food isolate | NSRC |
| 154326 | 2014 food isolate | NSRC |
| 154400 | 2014 food isolate | NSRC |
| *S. Infantis* 119944 *fnr* | *fnr* deletion mutant | this study |
| *S. Infantis* 119944 *fur* | *fur* deletion mutant | this study |
| *S. Infantis* 119944 *arcA* | *arcA* deletion mutant | this study |
| *S. Infantis* 119944 *arcB* | *arcB* deletion mutant | this study |
| *S. Infantis* 119944 *phoP* | *phoP* deletion mutant | this study |
| *S. Infantis* 119944 *ompR* | *ompR* deletion mutant | this study |
| *S. Infantis* 119944  *oxyR* | *oxyR* deletion mutant | this study |
| *S. Infantis* 119944 *soxR* | *soxR* deletion mutant | this study |
| *S. Infantis* 119944 *lrp* | *lrp* deletion mutant | this study |
| *S. Infantis* 119944 *rpoS* | *rpoS* deletion mutant | this study |
| *S. Infantis* 119944 *invA* | *invA* deletion mutant | this study |
| *S. Infantis* 119944 *traA* | *traA* deletion mutant | this study |
| *S. Infantis* 119944 *traB* | *traB* deletion mutant | this study |
| *S. Infantis* 119944 *traC* | *traC* deletion mutant | this study |
| *S*. Infantis 119944 *fnr/*pWSK29*::fnr* | *S*. Infantis 119944 *fnr*/ pWSK29::*fnr* | this study |
| *S*. Infantis 119944 *traB/*pWSK29*::traB* | *S*. Infantis 119944 *traB*/ pWSK29::*traB* | this study |
| **plasmids** |  |  |
| pKD46 |  | ([5](#_ENREF_5)) |
| pKD3 |  | ([5](#_ENREF_5)) |
| pCP20 |  | ([5](#_ENREF_5)) |
| pWSK29 |  | ([6](#_ENREF_6)) |
| pWSK29*::fnr* |  | this study |
| pWSK29*::traB* |  | this study |

NSRC – National *Salmonella* Reference Center Ministry of Health Israel.

**References**

1. **Aviv G, Tsyba K, Steck N, Salmon-Divon M, Cornelius A, Rahav G, Grassl GA, Gal-Mor O.** 2014. A unique megaplasmid contributes to stress tolerance and pathogenicity of an emergent Salmonella enterica serovar Infantis strain. Environmental microbiology **16:**977-994.

2. **Woodall LD, Russell PW, Harris SL, Orndorff PE.** 1993. Rapid, synchronous, and stable induction of type 1 piliation in Escherichia coli by using a chromosomal lacUV5 promoter. Journal of bacteriology **175:**2770-2778.

3. **Yi H, Cho YJ, Yong D, Chun J.** 2012. Genome sequence of Escherichia coli J53, a reference strain for genetic studies. Journal of bacteriology **194:**3742-3743.

4. **Brown NF, Vallance BA, Coombes BK, Valdez Y, Coburn BA, Finlay BB.** 2005. Salmonella pathogenicity island 2 is expressed prior to penetrating the intestine. PLoS pathogens **1:**e32.

5. **Datsenko KA, Wanner BL.** 2000. One-step inactivation of chromosomal genes in Escherichia coli K-12 using PCR products. Proceedings of the National Academy of Sciences of the United States of America **97:**6640-6645.

6. **Wang RF, Kushner SR.** 1991. Construction of versatile low-copy-number vectors for cloning, sequencing and gene expression in *Escherichia coli*. Gene **100:**195-199.
