# Supplementary material for: Horizontal Transfer of the Salmonella enterica Serovar Infantis Resistance and Virulence Plasmid pESI to the Gut Microbiota of Warm-Blooded Hosts
Source: mBio. 2016 Sep 6;7(5):e01395-16. doi: 10.1128/mBio.01395-16 (PMC5013300; doi:10.1128/mBio.01395-16)
Supplement: Table S2 — Primers used in this study. [file mbo004162973st2.docx]

**Table S2. Primers used in this study.**

| **Primer name** | **Sequence (5' to 3') *** | **Purpose** |
| --- | --- | --- |
| hp pESI Fw | GCGGTGAAGATGGTTATCAG | pESI backbone distribution |
| hp pESI Rv | GTGGTAGTTTGTCCTTTGGC |  |
| tcf ko Fw | AAACATGGTGTGGGCGTAAG | *tcf* operon distribution |
| tcf seq 12 |  |  |
| traC Fw | CCAGTATTTCCCGGCCTTCG | *tra* operon distribution |
| traC Rv | GTTATCCGGCGTGCAGAAAC |  |
| faeAB Fw | GAATTCTTTACCGGTAACGTTAG | k88- *fae* operon distribution |
| faeAB Rv | CTTCCACCAGTAACCAGAAATG |  |
| fim ko check Rv | CAATAATACTTTCTGTCAGATAAC | *ipf* operon distribution |
| fim chaperone Fw | GTATCAATCCGGGCATGATAAC |  |
| irp2 Fw | ACGGACATGCGTTATCAGTG | *ybt* distribution |
| irp2 Rv | GTTCCAGAGCGTGACCTGA |  |
| int F | GGCATCCAAGCAGCAAGC | class I integron distribution |
| int R | AAGCAGACTTGACCTGAT |  |
| dhpsF | TCTAGAATTCGTTCAAGCCGACGCCGCTTC | *sulI* distribution |
| dhpsR | GAGCTCGGCCGGAAGGTGAATGCTAGG |  |
| qacFclon | TCTAGAGCGCAGATCAGTTGGAAG | *qacE∆1* distribution |
| qacRclon | GAATTCAATGCCGAACACCGTCACC |  |
| MerA Fw | CAGTGGAAGTCCAGTACGGAG | *mer* operon distribution |
| MerA Rv | CTGGGCAGAAACGACAAGAC |  |
| tetAFII | GGCCTCAATTTCCTGACG | *tetA* distribution |
| tetAR2 | GGCATAGGCCTATCGTTTCCA |  |
| lacZ Fw | CGTTAACTCGGCGTTTCATCTG | *lacZ* presence |
| lacZ Rv | CAACCACCGCACGATAGAGATTC |  |
| invA Fw | TTCACTGACTTGCTATCTGC | *invA* presence |
| invA Rv | TCAGGAAACAAAACATATGC |  |
| RT PilV Fw | GGTGCATTACGTTCCTGGTC | RT-PCR of *pilV* |
| RT PilV Rv | GATCCCATATCAATGGCCGTG |  |
| RT k88 Fw | GCGTCATACGGGTATGGTCT | RT-PCR of *faeD* |
| RT k88 Rv | GGTACTTCGCCGTGGCATAC |  |
| rpoD Fw | GGTCTGACCATCGAACAGGTG | RT-PCR of *rpoD* |
| rpoD Rev | ATCAGACCGATGTTGCCTTC |  |
| RT 16S rRNA Fw | GGTTAAGTCCCGCAACGAG | RT-PCR of 16S *rRNA* |
| RT 16S rRNA Rv | CTTCTCTTTGTATGCGCCATTG |  |
| IncP Fw | CACTCTGCCCCTCAAGTGTC | pESI backbone distribution |
| IncP Rv | CAGGGAAATTCTCGTCCTTGC |  |
| yscR SPA seq F | CAACACCACCGGCATAGACG | *ssaR* distribution |
| yscR SPA seq R | TTCCAGGTCGTGTCATGAG |  |
| 8F 16S rRNA | AGAGTTTGATCCTGGCTCAG | identify mice cultured taxa |
| 338R 16S rRNA | TGCTGCCTCCCGTAGGAGT |  |
| Fnr Fw | GTATTGTTGTTCCCTGCCATCG | *fnr* deletion mutant |
| cm 5-Fnr (Rv) | GAAGCAGCTCCAGCCTACACACATAGGTCTGCTCAAGCCG |  |
| cm 3-Fnr (Fw) | CTAAGGAGGATATTCATATGGCTTAAACGTCAGCTAAACCATTG |  |
| Fnr Rv | GAAATCGTAAATAGGCAGGAAAGC |  |
| Fur Fw | ATCTGCGCCGCATCAATAG | *fur* deletion mutant |
| cm 5-Fur (Rv) | GAAGCAGCTCCAGCCTACACAGTCAGTCATGCGGAATCTGTC |  |
| cm 3-Fur (Fw) | CTAAGGAGGATATTCATATGGTGTAAATCTTTCGAAGAGCCAAC |  |
| Fur Rv | GTTGCGCCTGTGAATAAAAGG |  |
| arcA Fw | GATTTCATCAAACTGTTAACGTGCTAC | *arcA* deletion mutant |
| cm 5-arcA (Rv) | GAAGCAGCTCCAGCCTACACACATGTTTGCTACCTAAATTGCCA |  |
| cm 3-arcA (Fw) | CTAAGGAGGATATTCATATGCAGGATTAATTCGGCCCAGG |  |
| arcA Rv | CTTTGAAACCTCACGTCCTGTG |  |
| arcB Fw | ACGGCAGGTGAGATGATTCG | *arcB* deletion mutant |
| cm 5-arcB (Rv) | GAAGCAGCTCCAGCCTACACACATAGAGGATTCCTTCACCACA |  |
| cm 3-arcB (Fw) | CTAAGGAGGATATTCATATGGAAAGCCTGGGTGGCAAAC |  |
| arcB Rv | CATCCGCTGAGCACTACGC |  |
| Lrp Fw | CTGTGTTATGTATGTGCTGCATAATC | *lrp* deletion mutant |
| cm 5-Lrp (Rv) | GAAGCAGCTCCAGCCTACACAGTCTCTCTGTATTCCTTCCCTACTC |  |
| cm 3-Lrp (Fw) | CTAAGGAGGATATTCATATGCACGGAACAGGTGCAAAATC |  |
| Lrp Rv | GGGTTAAAGCTCAACAAGGCC |  |
| rpoS Fw | CGATGATTACCTGAGTGCCTAC | *rpoS* deletion mutant |
| cm 5-rpoS (Rv) | GAAGCAGCTCCAGCCTACACACATAAGGTGGCTCCTACCCG |  |
| cm 3-rpoS (Fw) | CTAAGGAGGATATTCATATGCGCTGTTCCGCGAGTAAGTA |  |
| rpoS Rv | CCGATGATTTGTCCACGCTG |  |
| oxyR Fw | GTAGCGCCATCAGGCAATAC | *oxyR* deletion mutant |
| cm 5-oxyR (Rv) | GAAGCAGCTCCAGCCTACACACATTATTCATCCTCCGTCGC |  |
| cm 3-oxyR (Fw) | CTAAGGAGGATATTCATATG GTTTAAGCCGTTCAACGCC |  |
| oxyR Rv | GCAGTTAACGGCAATGAAAG |  |
| soxR Fw | GGCTGTCTCCTGTAAGGTATG | *soxR* deletion mutant |
| cm 5-soxR (Rv) | GAAGCAGCTCCAGCCTACACACACCTAAAACTAAAGCGCCGC |  |
| cm 3-soxR (Fw) | CTAAGGAGGATATTCATATGCGTCTGCCCGCGTATCGTCAG |  |
| soxR Rv | GACTTTGGTACCGGCTATTCG |  |
| ompR Fw | GAGCTCTTGTTTGAGTGTTTCG | *ompR* deletion mutant |
| cm 5-ompR (Rv) | GAAGCAGCTCCAGCCTACACACATTGTCTGTACTCCCAAAGG |  |
| cm 3-ompR (Fw) | CTAAGGAGGATATTCATATGCTCGCCGCGAAGTTCATTTG |  |
| ompR Rv | CATCTGGTGGCTTAAGAATTCG |  |
| phoP Fw | GTGTTGATGCCGAAGGAATG | *phoP* deletion mutant |
| cm 5-phoP (Rv) | GAAGCAGCTCCAGCCTACACACATCTCTTCTCCCTTGTGTTAAC |  |
| cm 3-phoP (Fw) | CTAAGGAGGATATTCATATGGCGGGTTCGTTTTTTGCTG |  |
| phoP Rv | GAATGCTTTTAATCAGCCAGGG |  |
| TraA Fw | GACTCGGTGCGTTATACTAAGTAC | *traA* deletion mutant |
| cm 5-TraA (Rv) | GAAGCAGCTCCAGCCTACACACATAGCAAAGGTCTCCGATG |  |
| cm 3-TraA (Fw) | CTAAGGAGGATATTCATATGGAACAGGTGTCCCCATTAAAG |  |
| TraA Rv | CGGTGATTTGGCGAACTCAC |  |
| TraB Fw | CTCTGAACTGACGTGCTGATG | *traB* deletion mutant |
| cm 5-TraB (Rv) | GAAGCAGCTCCAGCCTACACACACGAAGTTCAACGCTCCG |  |
| cm 3-TraB (Fw) | CTAAGGAGGATATTCATATGCCAGTATTTCCCGGCCTTCG |  |
| TraB Rv | GATGTTCTGTCGTGGTCATGC |  |
| TraC Fw | CCAGTATTTCCCGGCCTTCG | *traC* deletion mutant |
| cm 5-TraC (Rv) | GAAGCAGCTCCAGCCTACACAGGTCATGCGAATGTCCTACTG |  |
| cm 3-TraC (Fw) | CTAAGGAGGATATTCATATGGTGTAATCCCCGACATACCC |  |
| TraC Rv | GTTATCCGGCGTGCAGAAAC |  |
| 1 invA Fw | GTGCTGCTTTCTCTACTTAACAG | *invA* deletion mutant |
| Cm 5-invA (Rv) | GAAGCAGCTCCAGCCTACACACATCGGCTTCAATCAAGATAAG |  |
| Cm 3-invA (Fw) | CTAAGGAGGATATTCATATGCCAATGGCGGCGAATTACGAG |  |
| 1 invA Rv | CTTGCTATCTGCTATCTCACCG |  |
| Cm 5 | TGTGTAGGCTGGAGCTGCTTC | chloramphenicol cassette creation |
| Cm 3 | CATATGAATATCCTCCTTAG |  |
| Fnr xhoI Fw | TTTTCTCGAGGGCAAAAAGTGCCTTATCGGTC | *fnr* complementation |
| Fnr xbaI Rv | TTTTTCTAGATTAAGCGACGTTGCGGGT |  |
| TraB XhoI Fw | TTTTCTCGAGTGCCTCCTCACTGATACCTCT | *traB* complementation |
| TraB xbaI Rv | TTTTTCTAGACTATGGGATGTTTACAAGGCGCTC |  |

* Nucleotide sequence from lambda-red recombinase antibiotic resistant cassettes and restriction enzyme sites added to the primers are underlined.
