# Supplementary material for: Horizontal Transfer of the Salmonella enterica Serovar Infantis Resistance and Virulence Plasmid pESI to the Gut Microbiota of Warm-Blooded Hosts
Source: mBio. 2016 Sep 6;7(5):e01395-16. doi: 10.1128/mBio.01395-16 (PMC5013300; doi:10.1128/mBio.01395-16)
Supplement: Table S3 — Mouse microbiota isolates found positive for pESI. [file mbo004162973st3.docx]

**Table S3. A list of mouse microbiota isolates that were found positive for pESI**

| **Days post infection** | **species** | **Isolate number** | **Mouse number** | **Sm-pretreated + / -** |
| --- | --- | --- | --- | --- |
| 6 | *E. coli* | 478-26 | 478 | + |
| 6 | *E. coli* | 481-49 | 481 | + |
| 6 | *E. coli* | 481-55 | 481 | + |
| 30 | *Ruminococcaceae* | 481-40 | 481 | + |
| 30 | *Ruminococcaceae* | 481-45 | 481 | + |
| 30 | *Ruminococcaceae* | 482-57 | 482 | + |
| 30 | Uncultured *Lactobacillus* | 482-52 | 482 | + |
| 30 | Uncultured *Lactobacillus* | 4482-5 | 482 | + |
| 30 | Uncultured *Lactobacillus* | 482-51 | 482 | + |
| 30 | *Lactobacillus* *reuteri* * | 482-54 | 482 | + |
| 30 | Uncultured *Lactobacillus* | 482-66 | 482 | + |
| 30 | Uncultured *Lactobacillus* | 482-49 | 482 | + |
| 30 | *Lactobacillus* *reuteri* * | 484-39 | 484 | - |
| 30 | *Lactobacillus* *reuteri* | *484-15* | 484 | - |
| 30 | *Lactobacillus* *reuteri* | 482-67 | 482 | + |
| 30 | *Ruminococcaceae* | 482-50 | 482 | + |
| 37 | *Lactobacillus* *reuteri* * | 484-32 | 484 | - |
| 51 | *Lactobacillus* *reuteri* | 477-52 | 477 | - |
| 51 | *Lactobacillus* *reuteri* | 477-45 | 477 | - |
| 51 | *Lactobacillus* *reuteri* | 477-33 | 477 | - |
| 51 | *Ruminococcaceae* | 477-42 | 477 | - |
| 65 | *Ruminococcaceae* | 477-56 | 477 | - |
| 72 | *Ruminococcaceae* | 477-61 | 477 | - |
| 86 | Uncultured *Lactobacillus* | 478-50 | 478 | + |
| 96 | *Lactobacillus* *reuteri* * | 480-44 | 480 | - |
| 107 | *Lactobacillus* *reuteri* | 478-9 | 478 | + |
| 107 | Uncultured bacterium | 481-27 | 481 | + |
| 107 | Uncultured bacterium | 481-29 | 481 | + |
| 107 | Uncultured bacterium | 478-25 | 478 | + |
| 121 | Uncultured bacterium | 478-10 | 478 | + |
| 121 | *Lactobacillus* *reuteri* * | 482-46 | 482 | + |
| 121 | Uncultured *Lactobacillus* | 478-17 | 478 | + |
| 121 | *Lactobacillus* *reuteri* | 499-8 | 499 | - |

*** Sequenced strains**
